# Serum metabolomics analysis of deficiency pattern and excess pattern in patients with rheumatoid arthritis

**DOI:** 10.1186/s13020-022-00632-5

**Published:** 2022-06-15

**Authors:** Bin Liu, Hongtao Guo, Li Li, Qi Geng, Ning Zhao, Yong Tan, Zhixing Nie, Guilin Ouyang, Aiping Lu, Cheng Lu

**Affiliations:** 1 Institute of Basic Research in Clinical Medicine, China Academy of Chinese Medical Sciences, Dongcheng District, Beijing, 100700 China; 2 The First Affiliated Hospital of Henan University of Chinese Medicine, Zhengzhou, China; 3 Shanghai Guanghua Hospital of Integreted Tranditional Chinese and Western Medicine, Shanghai, China; 4 School of Chinese Medicine, Hong Kong Baptist University, Hong Kong, China

**Keywords:** Serum metabolomics, Rheumatoid arthritis, Deficiency pattern, Excess pattern

## Abstract

**Background:**

Rheumatoid arthritis (RA) is a chronic and refractory autoimmune disease. Deficiency pattern (DP) and excess pattern (EP), as crucial types of Chinese medicine pattern diagnoses published by International Classification of Diseases 11th Revision (ICD-11), could provide new strategies for RA diagnosis. However, the biological basis of DP and EP of RA is not explicit.

**Methods:**

19 female RA DP patients, 41 female RA EP patients and 30 female healthy participants were included in the study. The serums of participants were collected and analyzed by metabolomics based on ultra-performance liquid chromatography coupled with quadrupole time-of-flight mass spectrometry to profile metabolic characteristics of RA DP and EP. Furthermore, bioinformatics analysis results were obtained by using Ingenuity Pathway Analysis (IPA) and statistical analysis was performed by SAS version 9.4 for further identification of potential biomarkers.

**Results:**

Serum metabolic profiling revealed 25 and 24 differential metabolites in RA DP and EP respectively, and 19 metabolites were common to RA DP and EP. Compared with DP group, L-Homocysteic acid, LysoPE(P-16:0/0:0), N(omega)-Hydroxyarginine and LysoPC(16:0/0:0) decreased (P < 0.05), and Pyruvic acid, D-Ribose, Gamma-Glutamylserine, PE(22:0/24:1(15Z)), Inosinic acid increased (P < 0.05) in EP group. Menawhile, S-Nitrosoglutathione, 5-Thymidylic acid, SN38 glucuronide, PE(22:0/24:0), PC(24:0/24:1(15Z)) and Bisdiphosphoinositol tetrakisphosphate increased significantly in DP group compared to EP group (P < 0.05). For the unique metabolites, bioinformatics analysis results showed that 5-Methoxytryptamine involved in Melatonin Degradation II and Superpathway of Melatonin Degradation is the key metabolite to RA DP. Meanwhile, GABA is the key metabolite in EP group, which involved in Glutamate Dependent Acid Resistance, GABA Receptor Signaling, Glutamate Degradation III (via 4-aminobutyrate) and 4-aminobutyrate Degradation I. Bioinformatics analysis between unique metabolites of RA DP and EP groups with human target genes for RA showed that 5-methoxytryptamine and LysoPC(18:1(9Z)/0:0), the unique metabolites of RA DP, might participate in colorectal cancer metastasis signaling, tumor microenvironment pathway, apoptosis signaling, MYC mediated apoptosis signaling, erythropoietin signaling pathway and LXR/RXR activation. Simultaneously, GABA, LysoPA(18:1(9Z)/0:0) and L-Targinine, the unique metabolites of RA EP, might participate in neuroinflammation signaling pathway, osteoarthritis pathway, glucocorticoid receptor signaling, ILK signaling, IL-17 signaling and HIF1*α* signaling.

**Conclusions:**

The study indicates that serum metabolomics preliminarily revealed the biological basis of RA DP and EP. 5-methoxytryptamine, LysoPC(18:1(9Z)/0:0) and GABA, LysoPA(18:1(9Z)/0:0), L-Targinine might be the predictors to distinguish the DP and EP of RA respectively. These interesting results provide thoughts for further study of traditional medicine patterns of ICD-11. It also contributes to provide strategy for personalized precision treatment of RA and further validation is needed.

## Background

Rheumatoid arthritis (RA) is a heterogeneous and refractory disease, which primarily involves the joints and cause disability [1]. The etiology of RA is thought to be associated with genetic and epigenetic components, environmental factors and autoimmunity [2]. Accordingly, new strategies and therapies are urgently required for the reason that many patients still do not respond to current therapies [1]. Chinese medicine (CM) is a system that uses an individualized approach based on the unique combination of symptoms and signs of each patient over 3000 years [3, 4]. CM pattern diagnosis is an approach that takes into account a broad spectrum of symptoms and signs and will lead more scientific new strategies and therapies for RA [3, 4].

Deficiency pattern (DP) and excess pattern (EP) are two crucial types of CM pattern diagnoses, which belongs to the principle-based patterns in the category of traditional medicine patterns published by International Classification of Diseases 11th Revision (ICD-11) [5–7]. Pattern differentiation in CM can help define specific indications for biomedical therapy in the disease treatment [8]. Based on the traditional medicine theory, principle-based patterns are an approach that take into account a wide range of signs and symptoms [4]. DP is manifested as pale or sallow complexion, bradypsychia, fatigue, shortness of breath, loose stools, uracratia, tongue with little or no coating, weak pulse, and others. Whereas EP is shown in red complexion, dysphoria, delirium, stentorian and raucous, constipation, dysuria, tongue coating thick and greasy, forceful pulse, and so on [9]. In CM clinical practice, patients with the same disease can be divided into different groups according to traditional medicine patterns [10]. In the near future, CM pattern diagnoses will facilitate further individualized medicine.

Metabolomics, the analysis of concentration profiles of low molecular weight metabolites present in biological fluids, has immense potential as a novel diagnostic tool in complex disorders such as RA, systemic lupus erythematosus [11, 12]. Simultaneously, metabolomics has highly discriminatory for biological perturbations or disease states in identifying new biomarkers [12]. Thus, metabolomics can be used to identify biological characteristics of CM pattern, which is an important approach to distinguish disease states. The urine metabolomics of diabetes mellitus (DM) indicated that xylose and C4 sugar 2 were higher in the EP than in the DP. These potential biomarkers reflected the deregulation of glucose metabolism in diabetic individuals that might be helpful for the diagnosis and therapy of DM [13]. Xylopyranoside for the diagnosis of DP and ribonic acid, uric acid, d-Ribose, and cyclohexanone for the diagnosis of EP in patients with chronic hepatitis B by urinary metabolomics. Meanwhile, the immune function of EP was demonstrated higher than that of DP [14]. The difference could help us transform the concept of DP and EP to modern therapeutic approaches and prompts us to further precision medicine.

Previous studies have studied the biological basis of cold pattern and heat pattern of RA by using plasma metabolomics [15, 16]. Meanwhile, the characteristic gene expression profile of DP in RA patients was explored by bioinformatics analysis approach [17], and the DP-associated autoantibodies was identified by using protein chips [18]. The above findings established the foundation to study CM pattern of RA. Therefore, this study will use serum metabolomics to explore the biological characteristics of the DP and EP in RA.

## Materials and methods

### Participants

The participants including female RA DP patients, female RA EP patients and healthy females were recruited from November 2018 to June 2019 in The First Affiliated Hospital of Henan University of CM. The sample size was based on previous studies [14, 19, 27]. The inclusion criteria include the following: (1) Patients with diagnosed of the RA and the diagnostic criteria followed 1987 or 2010 RA classification criteria [20, 21]; (2) DP and EP were classified according to the guidelines for the diagnosis and treatment of TCM patterns in RA [22], manifestations and other diagnostic information were determined independently by two experienced traditional Chinese physicians to ensure an objective evaluation; (3) The participants ranged in age from 18 to 80; (4) Participants signed the informed consent. Exclusion criteria: (1) RA patients with severe diseases requiring glucocorticoids; (2) RA patients who have been taking drugs for a long time or have not stopped taking drugs within 1 week; (3) Patients with circulatory, respiratory, digestive, urogenital, hematopoietic system, central nervous system and other serious diseases; (4) Pregnant or breast-feeding patients; (5) Patients with unclear prior medication records; (6) RA patients with other TCM patterns. Meanwhile, this study was approved by the Ethics Committee at the Institute of Basic Research in Clinical Medicine, China Academy of Chinese Medical Sciences (No.201,608).

### Serum samples

Each blood sample collected in a fasting condition was left standing at room temperature for 30 min. Then blood samples were centrifuged for 20 min at 3000 g at 4 °C to obtain the serum and transferred into clean tubes. All serum samples were stored at − 80 °C until LC-MS analysis.

### Metabolic profiling

Chromatographic separation was performed on a 1.8 μm, 2.1 mm × 100 mm ACQUITY UPLC®HSS T3 column (Waters, USA) using an ACQUITY Ultra Performance LC system (Waters corp., Milford, MA, USA) equipped with a binary solvent delivery system, an auto-sampler, and high temperature column oven. The column was maintained at 30 °C. The flow rate was set at 0.4 ml/min. The sample injection volume was 1.2 µl. Solvent A was water mixed with 0.1% formic acid, and solvent B was acetonitrile mixed with 0.1% formic acid. The gradient elution programmes were: 0–0.5 min 5% B, 0.5–7 min 100% B, 7–8.6 min 100% B, 8.6–11.5 min 100% B, 11.5–11.6 min 5% B, 11.6–16 min 5% B.

Mass spectrometry detection was acquired on a quadrupole time-of-flight mass spectrometer (Waters Corp., Milford, MA, USA) equipped with an electrospray ion source. The positive ion mode detection: The desolvation gas was set at 600 L/h at a temperature of 350 °C. The cone gas was set at 37 L/h. The source temperature was set at 100 °C. The capillary and cone voltage were optimized at 1.5 kV and 40 V, respectively. Full scan spectra were performed between 50 and 1000 m/z with a 0.2 s scan time and a 0.1 s interscan delay. The negative ion mode detection: The desolvation gas was set at 800 L/h at a temperature of 500 °C. The cone gas was set at 50 L/h. The source temperature was set at 120 °C. The capillary and cone voltage were optimized at 2 kV and 40 V, respectively. Full scan spectra were performed between 50 and 1200 m/z with a 0.2 s scan time and a 0.1 s interscan delay.

### Data pre-processing, multivariate statistical analysis and metabolite identification

The raw serum LC-MS data was pre-processed using Waters Progenesis QI 2.0 software (Nonlinear Dynamics, Newcastle, U.K.). Progenesis QI can provide an accurate measurement of the compounds in the bio-samples and facilitate flexible comparisons across runs. It included steps of importing data, reviewing alignment, experiment design setup, picking peaks, identifying and reviewing compounds and performing compound statistical analysis. Then, the data were exported into SIMCA-P software (v13.0) (Umetrics, Malmö, Sweden) for multivariable statistical analysis (MVA). The MVA of principal component (PCA), partial least squares discriminant (PLS-DA) and orthogonal partial least square-discriminant (OPLS-DA) models were both applied to observe the classifications for different groups in score plots. Next, to find significant metabolites contributing to the classifications, ions with variable importance in the projection (VIP) > 1 were picked out from the OPLS-DA loading plots. Then univariate analysis of* t*-test and fold-change were also applied to these ions. Ions with *P*-values < 0.05, fold-changes > 2.0 (or < 0.5) and relative standard deviation (RSD) < 0.3 were finally regarded as differentiated metabolite ions. Then they were structurally identified and interpreted based on searches of their accurate masses in HMDB (www.hmdb.ca) database. Lastly, the isotopic distribution, retention time and fragments of commercial standards were further confirmed for the metabolites of interest.

### Bioinformatics analysis

The analysis of canonical pathways, bio-functions and the networks were conducted by using the Ingenuity Pathway Analysis system (IPA, http://www.ingenuity.com) for the candidate metabolites and proteins to gain further insights into the typical metabolic alterations.

### Statistical analysis

Continuous variables were described using mean ± standard deviation. The count data were expressed as the number of cases or percentages. Differences between groups were assessed using analysis of* t*-tests for continuous variables and the Chi-square test or Fisher’s exact test for categorical variables. SAS version 9.4 was used for all statistical analyses. *P* value was set as 0.05 for statistical significance.

## Results

### Clinical characteristics of participants

**Fig. 1:**
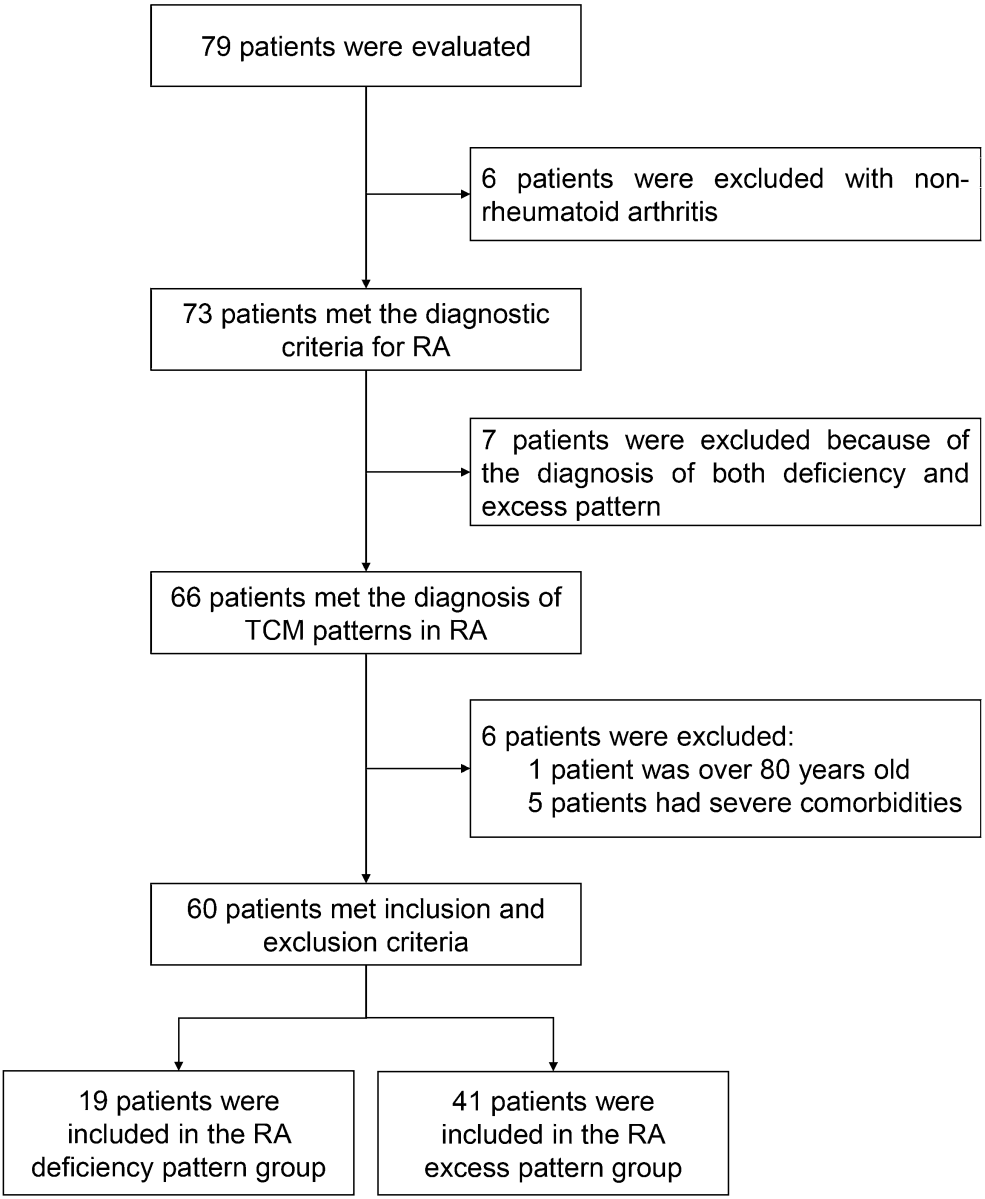
Flowchart of rheumatoid arthritis deficiency pattern and excess pattern groups

A total of 90 participants including 19 female RA DP patients, 41 female RA EP patients (Fig. 1) and 30 healthy females were recruited. The characteristics of the enrolled subjects, including age, duration of RA, body mass index (BMI), white blood cell (WBC), red blood cell (RBC), hemoglobin (HGB), platelet (PLT), albumin (ALB), erythrocyte sedimentation rate (ESR), C-reactive protein (CRP), rheumatoid factor (RF)-IgG, RF-IgA, RF-IgM, smoked, hypertension, diabetes and coronary heart disease were shown in Table 1. In terms of age, there was no statistical difference between DP, EP and healthy participants, respectively (*P* > 0.05), but there was statistical difference between DP and EP (*P* = 0.003). There were no significant differences in BMI, WBC, RBC, HGB, PLT, ALB, between the three groups (*P* > 0.05). In terms of duration of RA, ESR, CRP, RF-IgG, RF-IgA, RF-IgM, smoked, hypertension, diabetes and coronary heart disease, there were no statistically significant differences between DP and EP (*P* > 0.05).

**Table 1:** Clinical characteristics of participants

Indicators	Reference range	Deficiency pattern	Excess pattern	Healthy participants	P value
Number of participants	–	19	41	30	–
Age (years)	–	59.68 ± 6.83	50.95 ± 11.08	56.07 ± 8.41	0.003
Duration of RA (month)	–	152.45 ± 142.21	147.14 ± 131.31	–	0.162
BMI	18.5–23.9	22.20 ± 3.69	21.33 ± 2.94	20.86 ± 3.21	0.423
WBC (×10^9^/L)	4–10	6.85 ± 2.86	5.79 ± 1.85	6.50 ± 1.60	0.089
RBC (×10^12^/L)	3.5–5.5	3.90 ± 0.61	3.89 ± 0.34	4.02 ± 0.29	0.961
HGB (g/L)	110–160	106.00 ± 23.00	107.13 ± 14.66	109.77 ± 12.02	0.818
PLT (×10^9^/L)	100–300	275.00 ± 123.94	262.87 ± 70.85	243.23 ± 29.25	0.632
ALB (g/L)	40–60	37.86 ± 4.95	37.71 ± 4.21	39.10 ± 3.98	0.907
ESR (mm/h)	0–20	50.05 ± 29.34	46.78 ± 27.10	–	0.673
CRP (mg/L)	0–20	35.89 ± 34.04	28.21 ± 29.73	–	0.431
RF-IgG (RU/ml)	0–20	28.28 ± 65.63	15.35 ± 21.28	–	0.824
RF-IgA (RU/ml)	0–20	131.43 ± 107.41	120.47 ± 99.27	–	0.697
RF-IgM(RU/ml)	0–20	260.02 ± 136.80	292.86 ± 125.36	–	0.269
Smoked (n, %)	–	0 (0.0%)	2 (4.9%)	–	1.000*
Hypertension (n, %)	–	6 (31.6%)	16 (39.0%)	–	0.774*
Diabetes (n, %)	–	3 (15.8%)	6 (14.6%)	–	1.000*
Coronary heart disease (n, %)	–	7 (36.8%)	9 (22.0%)	–	0.347*

* Fisher’s exact test

### PCA and PLS-DA analysis of metabolomics profiles in DP, EP, and healthy groups

**Fig. 2:**
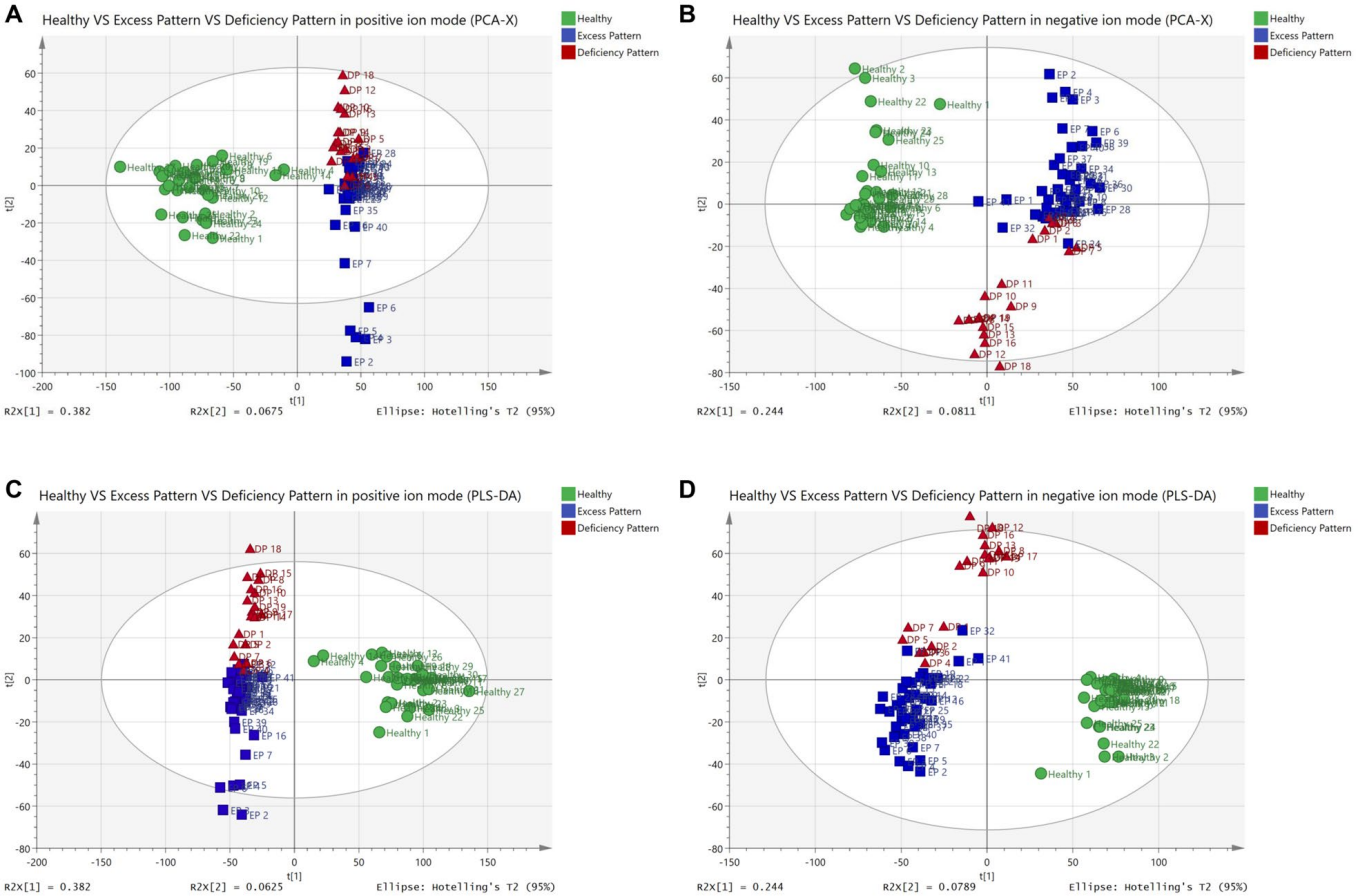
PCA and PLS-DA scores of healthy, RA DP and EP serum metabolic profile. **A** PCA score in positive ion mode. **B** PCA score in negative ion mode. **C** PLS-DA score in positive ion mode. **D** PLS-DA score in negative ion mode

PCA was used to determine the presence of inherent similarities in spectral profiles and the corresponding PLS-DA analysis was used to identify discriminating metabolites and differentiate the three groups. Although there was some overlap between DP and EP group, the PCA (Fig. 2A, B) and PLS-DA (Fig. 2C, D) results revealed a clear separation between the serum samples of participants from the DP, EP and healthy groups which demonstrating specific metabolic profiles of different groups. In PLS-DA positive ion mode: R^2^X = 0.471 (cum), R^2^Y = 0.862 (cum), Q^2^ = 0.739(cum). And in PLS-DA negative ion mode: R^2^X = 0.323 (cum), R^2^Y = 0.808 (cum), Q^2^ = 0.772 (cum).

### PCA and OPLS-DA analysis of metabolomics profiles in DP and healthy groups

**Fig. 3:**
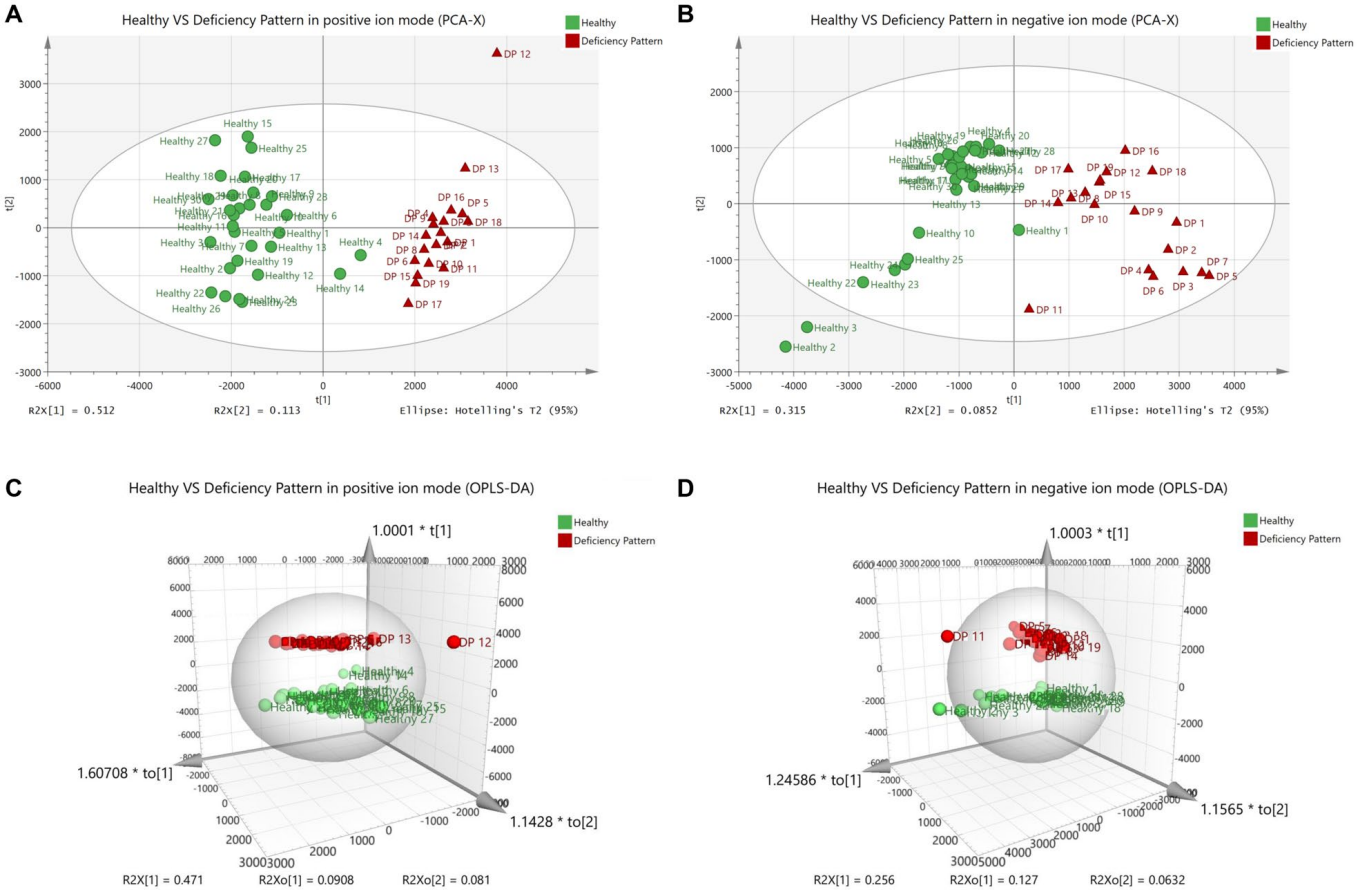
PCA and OPLS-DA scores of healthy and RA DP serum metabolic profile. **A** PCA score in positive ion mode. **B** PCA score in negative ion mode. **C** OPLS-DA score in positive ion mode. **D** OPLS-DA score in negative ion mode

PCA (Fig. 3A, B) and OPLS-DA (Fig. 3C, D) analysis results showed that DP group was clearly separated from those in the healthy group, indicating that the RA DP patients have totally different metabolic serum characteristics from healthy individuals. In OPLS-DA positive ion mode: R^2^X = 0.689 (cum), R^2^Y = 0.992 (cum), Q^2^ = 0.972 (cum). And in OPLS-DA negative ion mode: R^2^X = 0.446 (cum), R^2^Y = 0.99 (cum), Q^2^ = 0.972 (cum). By metabolite identification, 25 differential metabolites including 12 in positive ion mode and 13 in negative ion mode were obtained between DP and healthy groups (Table 2). Compared with the healthy group, 10 metabolites in DP group showed a downward trend, and 15 metabolites showed an upward trend (*P* < 0.01).

**Table 2:** Serum metabolites of RA DP patients

No.	Metabolite	m/z	RT (min)	Formula	VIP	Fold Change	Trend
1	L-Homocysteic acid	184.07	8.01	C_4_H_9_NO_5_S	3.93	− 2.25	↓
2	5-Methoxytryptamine	191.04	0.57	C_11_H_14_N_2_O	1.43	− 2.54	↓
3	4-Fumarylacetoacetic acid	182.96	0.48	C_8_H_8_O_6_	2.09	10.33	↑
4	S-Nitrosoglutathione	337.06	0.62	C_10_H_16_N_4_O_7_S	10.09	2194.14	↑
5	LysoPE(P-16:0/0:0)	438.30	8.35	C_21_H_44_NO_6_P	1.14	− 2.06	↓
6	LysoPC(16:0/0:0)	496.34	8.16	C_24_H_50_NO_7_P	32.88	− 2.01	↓
7	LysoPC(P-18:0/0:0)	508.38	8.55	C_26_H_54_NO_6_P	3.03	− 3.02	↓
8	LysoPC(18:1(9Z)/0:0)	522.36	8.37	C_26_H_52_NO_7_P	14.21	− 2.14	↓
9	LysoPC(20:1(11Z)/0:0)	550.39	8.87	C_28_H_56_NO_7_P	1.53	− 2.68	↓
10	Bisdiphosphoinositol tetrakisphosphate	820.81	0.55	C_6_H_20_O_30_P_8_	1.27	2.87	↑
11	PE(22:0/24:0)	888.80	0.55	C_51_H_102_NO_8_P	1.41	2.78	↑
12	PC(24:0/24:1(15Z))	956.79	0.55	C_56_H_110_NO_8_P	1.35	2.58	↑
13	Pyruvic acid	174.96	0.49	C_3_H_4_O_3_	5.03	12.87	↑
14	D-Ribose	130.97	0.49	C_5_H_10_O_5_	2.13	7.30	↑
15	N(omega)-Hydroxyarginine	224.98	0.63	C_6_H_14_N_4_O_3_	1.42	− 7.41	↓
16	Citric acid	191.02	0.64	C_6_H_8_O_7_	3.10	6.36	↑
17	Gamma-Glutamylserine	269.08	0.60	C_8_H_14_N_2_O_6_	4.22	41.27	↑
18	5-Thymidylic acid	357.03	0.63	C_10_H_15_N_2_O_8_P	4.62	2.49	↑
19	Inosinic acid	329.04	0.59	C_10_H_13_N_4_O_8_P	6.12	112.81	↑
20	SN38 glucuronide	549.05	0.64	C_28_H_28_N_2_O_11_	1.58	21.42	↑
21	Glucosylceramide (d18:1/12:0)	678.51	0.56	C_36_H_69_NO_8_	1.07	− 3.46	↓
22	PE(20:3(5Z,8Z,11Z)/22:6(4Z,7Z,10Z,13Z,16Z,19Z))	794.42	0.56	C_47_H_76_NO_8_P	1.96	− 3.95	↓
23	PC(O-22:1(13Z)/22:3(10Z,13Z,16Z))	878.77	0.48	C_52_H_98_NO_7_P	1.78	4.00	↑
24	PE(22:0/24:1(15Z))	866.78	0.53	C_51_H_100_NO_8_P	1.90	3.09	↑
25	Octanoyl-CoA	874.24	0.48	C_29_H_50_N_7_O_17_P_3_S	1.38	3.61	↑

Fold change refers to the “RA DP group vs. healthy group” change value

*LysoPE* Lysophosphatidylethanolamine;* LysoPC* Lysophosphatidylcholine;* PC* Phosphatidylcholine;* PE* Phosphatidylethanolamine

### PCA and OPLS-DA analysis of metabolomics profiles in EP and healthy groups

**Fig. 4:**
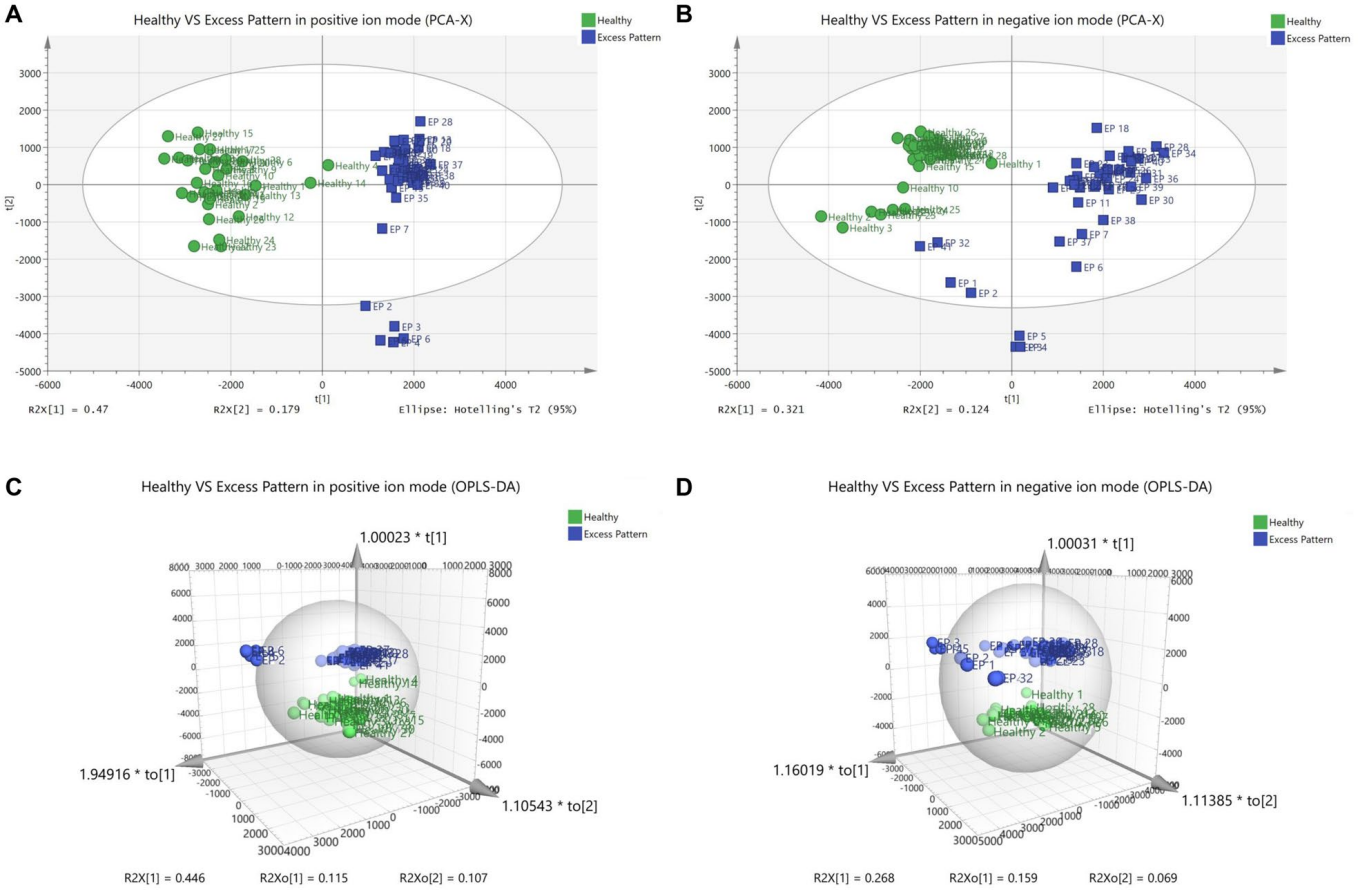
PCA and OPLS-DA scores of healthy and RA EP serum metabolic profile. **A** PCA score in positive ion mode. **B** PCA score in negative ion mode. **C** OPLS-DA score in positive ion mode. **D** OPLS-DA score in negative ion mode

PCA (Fig. 4A, B) and OPLS-DA (Fig. 4C, D) analysis results showed that EP group was clearly separated from those in the healthy group, indicating that the RA EP patients have totally different metabolic serum characteristics from healthy individuals. In OPLS-DA positive ion mode: R^2^X = 0.667 (cum), R^2^Y = 0.985 (cum), Q^2^ = 0.968 (cum). And in OPLS-DA negative ion mode: R^2^X = 0.496 (cum), R^2^Y = 0.983 (cum), Q^2^ = 0.965 (cum). By metabolite identification, 24 differential metabolites including 11 in positive ion mode and 13 in negative ion mode were obtained between DP and healthy groups (Table 3). Compared with the healthy group, 7 metabolites in DP group showed a downward trend, and 17 metabolites showed an upward trend (*P* < 0.01).

**Table 3:** Serum metabolites of RA EP patients

No.	Metabolite	m/z	RT (min)	Formula	VIP	Fold Change	Trend
1	Gamma-Aminobutyric acid	104.11	8.16	C_4_H_9_NO_2_	2.56	-2.28	↓
2	L-Homocysteic acid	184.07	8.01	C_4_H_9_NO_5_S	4.74	-2.72	↓
3	S-Nitrosoglutathione	337.06	0.62	C_10_H_16_N_4_O_7_S	10.03	2044.09	↑
4	LysoPA(18:1(9Z)/0:0)	459.25	8.01	C_21_H_41_O_7_P	1.01	-2.12	↓
5	LysoPE(P-16:0/0:0)	438.30	8.35	C_21_H_44_NO_6_P	1.40	-2.47	↓
6	LysoPC(16:0/0:0)	496.34	8.16	C_24_H_50_NO_7_P	38.02	-2.29	↓
7	LysoPC(P-18:0/0:0)	508.38	8.55	C_26_H_54_NO_6_P	3.19	-2.96	↓
8	DG(18:3(6Z,9Z,12Z)/22:6(4Z,7Z,10Z,13Z,16Z,19Z)/0:0)	685.44	11.50	C_43_H_66_O_5_	1.24	3.42	↑
9	Bisdiphosphoinositol tetrakisphosphate	820.81	0.55	C_6_H_20_O_30_P_8_	1.24	2.67	↑
10	PE(22:0/24:0)	888.80	0.55	C_51_H_102_NO_8_P	1.36	2.56	↑
11	PC(24:0/24:1(15Z))	956.79	0.55	C_56_H_110_NO_8_P	1.29	2.36	↑
12	Pyruvic acid	174.96	0.49	C_3_H_4_O_3_	4.84	15.21	↑
13	D-Ribose	130.97	0.49	C_5_H_10_O_5_	2.13	9.07	↑
14	L-Targinine	187.07	0.60	C_7_H_16_N_4_O_2_	1.68	53.79	↑
15	N(omega)-Hydroxyarginine	224.98	0.63	C_6_H_14_N_4_O_3_	1.26	-9.13	↓
16	Citric acid	191.02	0.64	C_6_H_8_O_7_	2.66	6.12	↑
17	Gamma-Glutamylserine	269.08	0.60	C_8_H_14_N_2_O_6_	4.21	52.50	↑
18	5-Thymidylic acid	357.03	0.63	C_10_H_15_N_2_O_8_P	3.53	2.17	↑
19	Inosinic acid	329.04	0.59	C_10_H_13_N_4_O_8_P	5.62	124.17	↑
20	SN38 glucuronide	549.05	0.64	C_28_H_28_N_2_O_11_	1.14	15.33	↑
21	PC(O-22:1(13Z)/22:3(10Z,13Z,16Z))	878.77	0.48	C_52_H_98_NO_7_P	1.63	4.20	↑
22	PE(22:0/24:1(15Z))	866.78	0.53	C_51_H_100_NO_8_P	1.91	3.57	↑
23	Octanoyl-CoA	874.24	0.48	C_29_H_50_N_7_O_17_P_3_S	1.21	3.57	↑
24	PC(24:0/24:0)	1002.76	0.53	C_56_H_112_NO_8_P	1.30	2.14	↑

Fold change refers to the “RA EP group vs. healthy group” change value

*LysoPA* Lysophosphatidic acid, * LysoPE* Lysophosphatidylethanolamine, * LysoPC* Lysophosphatidylcholine, * DG* Diacyl glycerol, * PE* Phosphatidylethanolamine, * PC* Phosphatidylcholine

### Comparative analysis of common differential metabolites between DP and EP groups

19 metabolites were common to DP and EP groups, including 8 in positive ion mode: L-Homocysteic acid, S-Nitrosoglutathione, LysoPE(P-16:0/0:0), LysoPC(16:0/0:0), LysoPC(P-18:0/0:0), Bisdiphosphoinositol tetrakisphosphate, PE(22:0/24:0), PC(24:0/24:1(15Z)), and 11 in negative ion mode: Pyruvic acid, D-Ribose, N(omega)-Hydroxyarginine, Citric acid, Gamma-Glutamylserine, 5-Thymidylic acid, Inosinic acid, SN38 glucuronide, PC(O-22:1(13Z)/22:3(10Z,13Z,16Z)), PE(22:0/24:1(15Z)), Octanoyl-CoA.

**Fig. 5:**
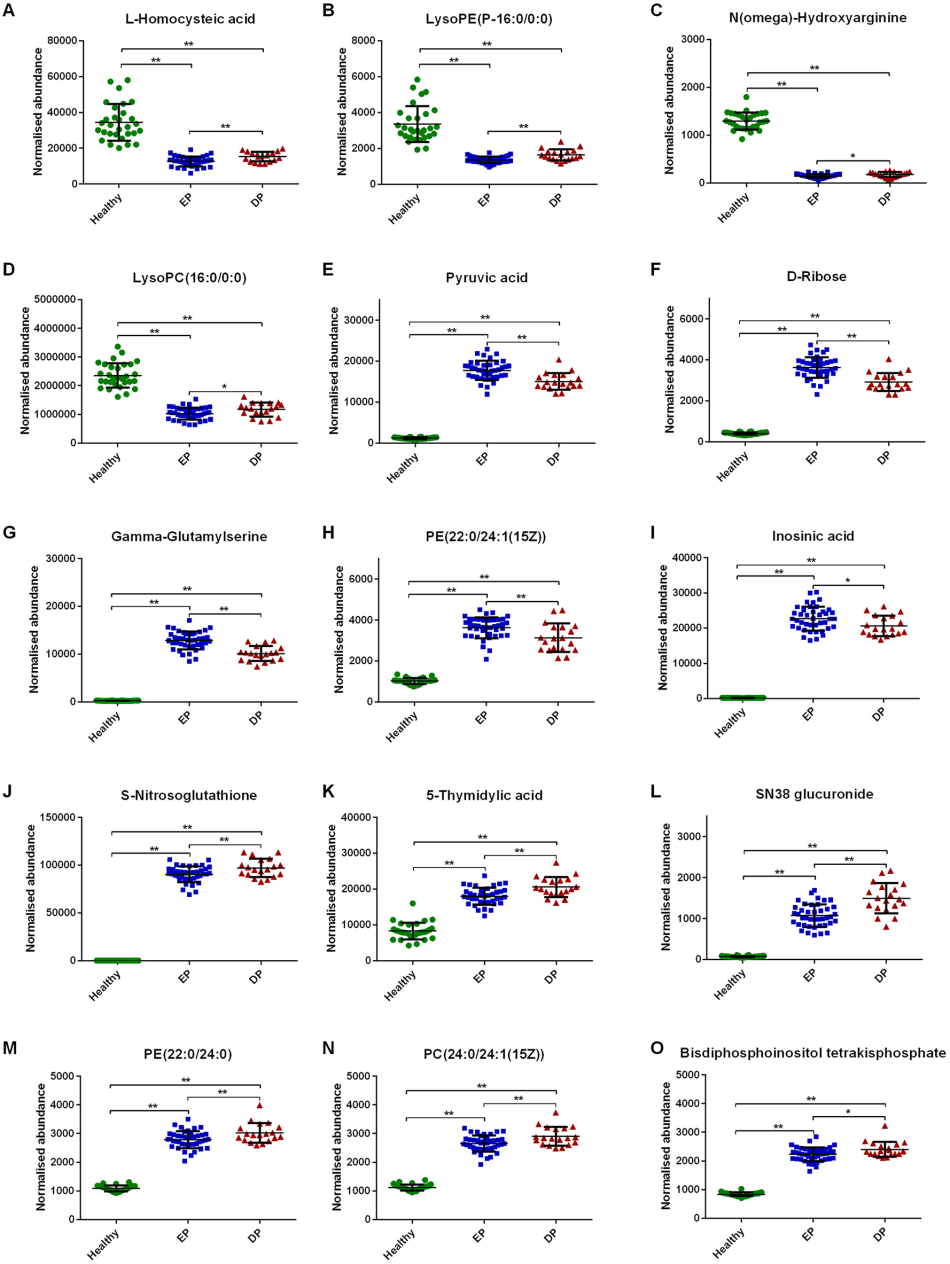
Comparative analysis of common differential metabolites between RA DP and EP groups. Compared with DP group, **A-–D** decreased and **E-I** increased in EP group. **J-O** increased in DP group compared to EP group. * refers to *P* < 0.05; ** refers to *P* < 0.01

**Fig. 6:**
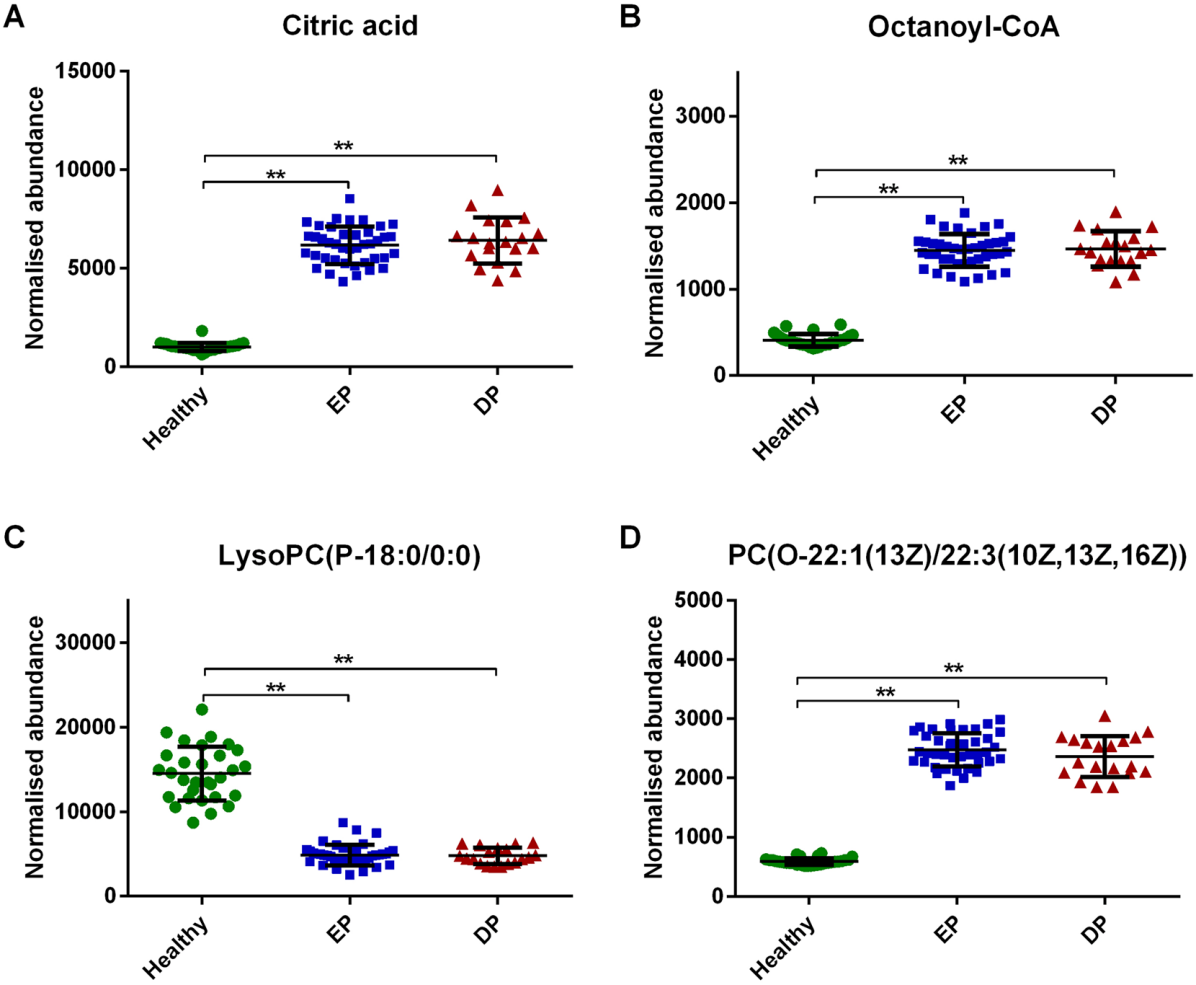
Comparative analysis of common differential metabolites between RA DP and EP groups. There was no statistical difference in **A-D** between DP and EP group. ** refers to P 0.01

Compared with DP group, L-Homocysteic acid (Fig. 5A), LysoPE(P-16:0/0:0) (Fig. 5B), N(omega)-Hydroxyarginine (Fig. 5C) and LysoPC(16:0/0:0) (Fig. 5D) decreased (*P* < 0.05), and Pyruvic acid (Fig. 5E), D-Ribose (Fig. 5F), Gamma-Glutamylserine (Fig. 5G), PE(22:0/24:1(15Z)) (Fig. 5H), Inosinic acid (Fig. 5I) increased (*P* < 0.05) in EP group. Menawhile, S-Nitrosoglutathione (Fig. 5J), 5-Thymidylic acid (Fig. 5K), SN38 glucuronide (Fig. 5L), PE(22:0/24:0) (Fig. 5M), PC(24:0/24:1(15Z)) (Fig. 5N) and Bisdiphosphoinositol tetrakisphosphate (Fig. 5O) increased significantly in DP group compared to EP group (*P* < 0.05). However, there was no statistical difference in Citric acid (Fig. 6A), Octanoyl-CoA (Fig. 6B), LysoPC(P-18:0/0:0) (Fig. 6C) and PC(O-22:1(13Z)/22:3(10Z,13Z,16Z)) (Fig. 6D) between DP and EP group (*P* > 0.05).

### Bioinformatics analysis of unique metabolites of DP and EP groups

**Fig. 7:**
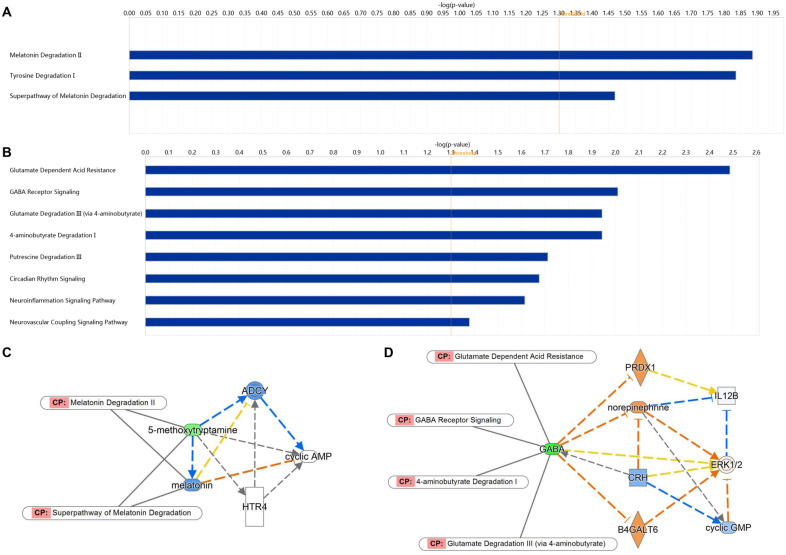
Bioinformatics analysis of unique metabolites of RA DP and EP groups. **A** Biological pathways related to RA DP; **B** Biological pathways related to RA EP; **C** Network analysis of key metabolites of RA DP; **D** Network analysis of key metabolites of RA EP. Threshold = 1.3

There were 6 unique metabolites in DP group: 5-Methoxytryptamine, 4-Fumarylacetoacetic acid, LysoPC(18:1(9Z)/0:0), LysoPC(20:1(11Z)/0:0), Glucosylceramide (d18:1/12:0), PE(20:3 (5Z,8Z,11Z) /22:6 (4Z,7Z,10Z,13Z,16Z,19Z)), and 5 unique metabolites in EP group: Gamma-Aminobutyric acid (GABA), LysoPA(18:1(9Z)/0:0), DG(18:3 (6Z,9Z,12Z)/22:6 (4Z,7Z,10Z,13Z,16Z,19Z) /0:0), L-Targinine, PC(24:0/24:0). IPA core function analysis results show that Melatonin Degradation II, Tyrosine Degradation I, Superpathway of Melatonin Degradation are biological pathways closely related to DP group (Fig. 7A). And Glutamate Dependent Acid Resistance, GABA Receptor Signaling, Glutamate Degradation III (via 4-aminobutyrate), 4-aminobutyrate Degradation I, Putrescine Degradation III, Circadian Rhythm Signaling, Neuroinflammation Signaling Pathway, Neurovascular Coupling Signaling Pathway are related biological pathways in EP group (Fig. 7B). In network analysis results, 5-Methoxytryptamine involved in Melatonin Degradation II and Superpathway of Melatonin Degradation is the key metabolite to DP group (Fig. 7C). Meanwhile, GABA is the key metabolite in EP group (Fig. 7D), which involved in Glutamate Dependent Acid Resistance, GABA Receptor Signaling, Glutamate Degradation III (via 4-aminobutyrate) and 4-aminobutyrate Degradation I.

### Bioinformatics analysis between unique metabolites of RA DP and EP groups with human target genes for RA

**Fig. 8:**
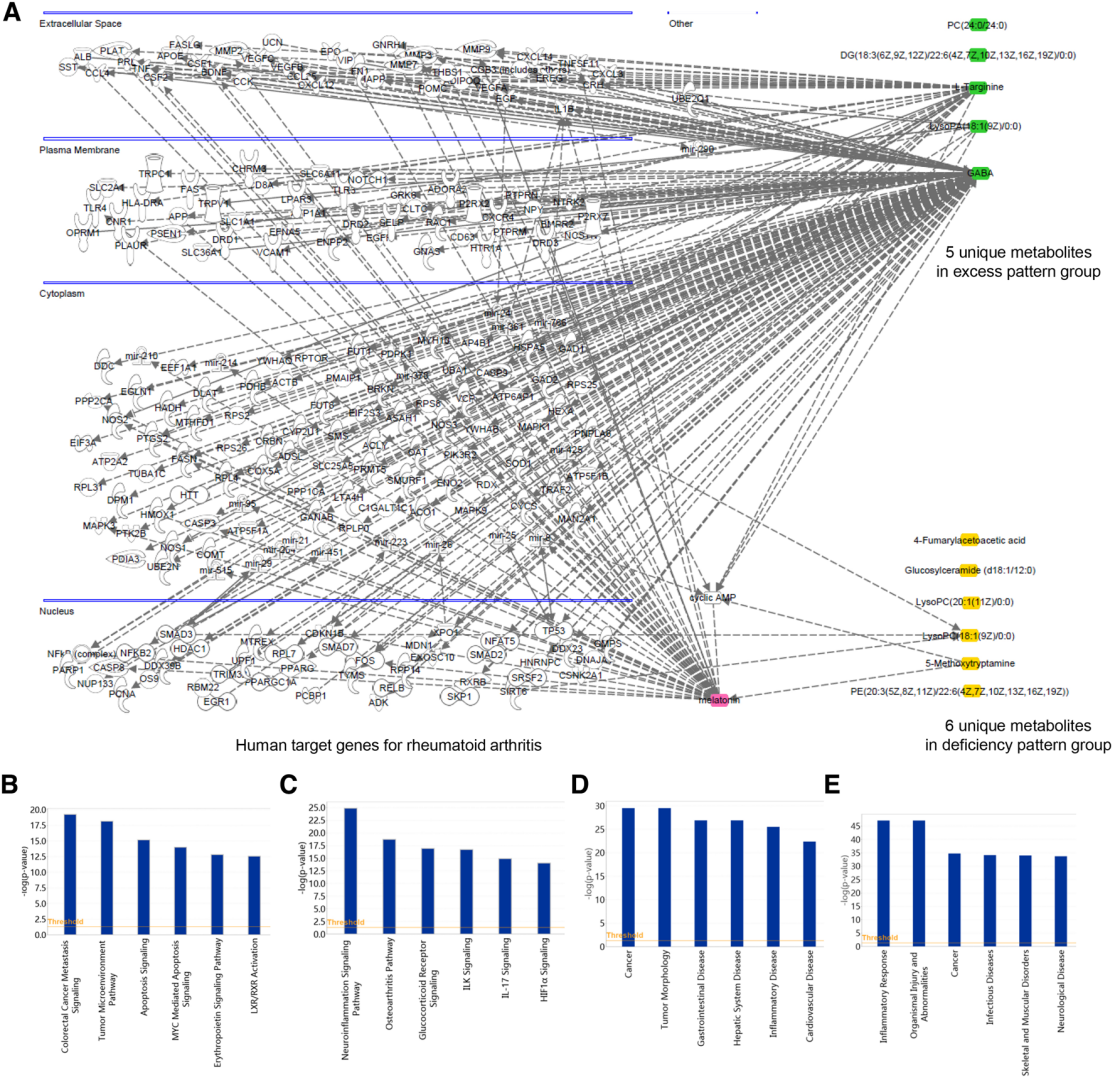
Bioinformatics analysis between unique metabolites of RA DP and EP groups with human target genes for RA. **A** The related networks between unique metabolites of RA DP and EP groups with human target genes for RA; The related biological pathways between unique metabolites of RA DP group (**B**) and EP group (**C**) with human target genes for RA; The related disease and functional analysis between unique metabolites of RA DP group (**D**) and EP group (**E**) with human target genes for RA. Threshold = 1.3

GeneCards, the human gene database, was used to retrieve RA human target genes. Through the IPA analysis between unique metabolites of RA DP and EP groups with human target genes for RA, the network relationship was obtained (Fig. 8A). Among the 6 unique metabolites of RA DP, 5-methoxytryptamine and LysoPC(18:1(9Z)/0:0) were closely related to RA target genes. In particular, melatonin can link 5-methoxytryptamine to target genes in the extracellular space, plasma membrane, cytoplasm, and nucleus that are related to RA. Furthermore, 5-methoxytryptamine might play a role in cancer, tumor morphology, gastrointestinal disease, hepatic system disease, inflammatory disease, cardiovascular disease (Fig. 8D) etc. through colorectal cancer metastasis signaling, tumor microenvironment pathway, apoptosis signaling, MYC mediated apoptosis signaling, erythropoietin signaling pathway, LXR/RXR activation (Fig. 8B) etc. Meanwhile, GABA, LysoPA(18:1(9Z)/0:0) and L-Targinine, the unique metabolites of RA EP, were closely related to RA target genes. GABA, in particular, has been linked to RA-related target genes found in the extracellular space, plasma membrane, cytoplasm, and nucleus. Through neuroinflammation signaling pathway, osteoarthritis pathway, glucocorticoid receptor signaling, ILK signaling, IL-17 signaling and HIF1*α* signaling (Fig. 8C) etc. GABA might participate in inflammatory response, organismal injury and abnormalities, cancer, infectious diseases, skeletal and muscular disorders, neurological disease (Fig. 8E) etc.

**Fig. 9:**
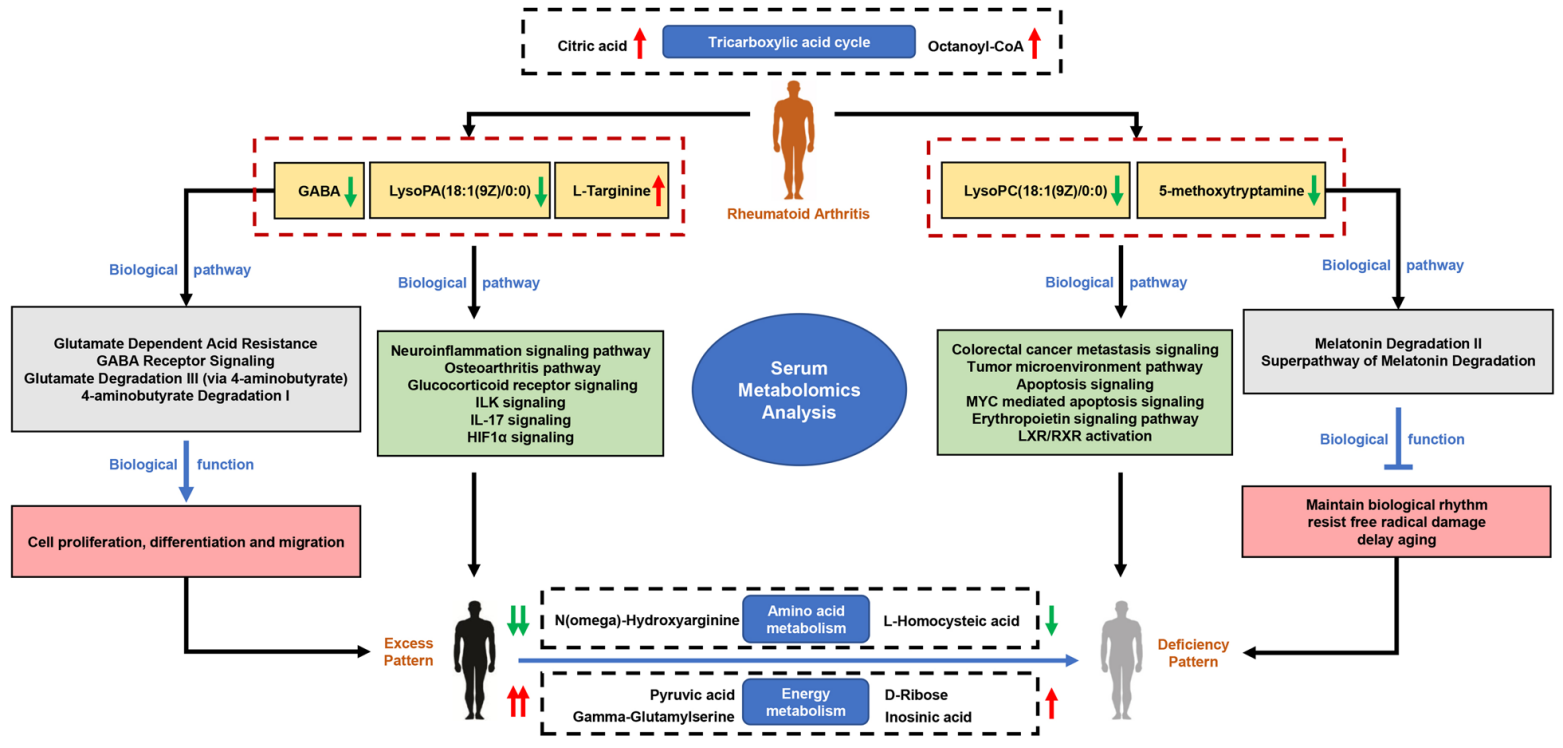
The potential serum metabolomics characteristics of DP and EP in patients with RA

## Discussions

CM is a medical system with at least 3000 years of uninterrupted clinical practice. With the release of ICD-11, traditional medicine, commonly used across many countries including China, Japan, and Korea, was brought within the perimeter of mainstream medicine to be considered right [23]. From global extension of traditional medicine to the integration of traditional medicine with multiple Western medicine-based disciplines, people all over the world will benefit [24]. Subgrouping of patients with complex diseases, such as RA, according to CM pattern diagnosis has the potential to provide opportunities for new strategies and better treatment outcomes [8, 25]. Moreover, metabolomics was used to explore the biological characteristics of DP and EP can provide scientific evidence for CM pattern diagnosis [5, 13, 14, 26].

RA is a heterogeneous and refractory inflammatory joint disease, which still require new strategies and therapies urgently [1]. CM pattern differentiation based on clinical signs and symptoms showed a diverse range of biomolecules, proteins and genes from RA patients correlated well with cold pattern and heat pattern [4]. Particularly, the cold pattern was related to Toll-like receptor signaling pathway, cell proliferation and the Jak-STAT cascade. The following related pathways in heat pattern were included: Calcium signaling pathway, cell adhesion molecules, PPAR signaling pathway, fatty acid metabolism and I-κB kinase/NF-κB cascade [27, 28]. Previous studies have shown that traditional Chinese medicine would be more effective in RA patients with DP, and western medicine would be more effective in RA patients with cold pattern. Based on different symptoms and signs, superior efficacy can be achieved in RA patients with different CM patterns [29]. These results suggest that a better understanding of CM patterns might help to choose the most appropriate therapies [30].

In the present study, metabonomic technology was used to stratify the DP and EP in RA patients. Although the age of patients with DP and EP has a statistical difference, RA is a chronic autoimmune disease characterized by immunomodulatory disorder and a failure of spontaneous resolution of inflammation [31]. Previous studies have shown that the human immune system deteriorates with age and immunosenescence is a series of age-related changes that affect the immune system [32]. As an autoimmune disease, the immune function of patients with RA may also be affected by age. In CM clinical practice, the age of patients with DP was higher than that of patients with EP [33]. This is consistent with our research. The results illustrate that substance fundaments and biological characteristics are separated clearly between DP and EP groups by metabolic profiles (Fig. 9). 19 differential metabolites in common were identified between DP and EP groups. There was no statistical difference between citric acid and octanoyl-COA in RA DP and EP groups. These two metabolites and their participation in the tricarboxylic acid cycle [34] are the potential biological characteristics of RA. Among the 15 statistical differences metabolites, L-Homocysteic acid and N(omega)-Hydroxyarginine decreased in EP is more obvious than that in DP. This suggests that amino acid metabolism is more significant in EP than in DP. Meanwhile, pyruvic acid, D-Ribose, inosinic acid and Gamma-Glutamylserine increased in EP is more obvious than that in DP. This reflects that energy metabolism is more significant in EP than in DP. In addition, the changes of lipid metabolites were different in DP and EP groups which indicates there was also lipid metabolism disorder in RA. These common metabolites have potential value for the identification of RA DP and EP.

Among the specific metabolites of RA DP, 5-methoxytryptamine as the intermediate of melatonin showed a decreasing trend. Melatonin secreted by the pineal gland widely exists in the body, which has the function of scavenging reactive oxygen species and active nitrogen and plays a certain role in hormone secretion, biological rhythm, anti-free radical damage and anti-aging [35, 36]. Previous study has found that the level of melatonin with Heart-spleen Deficiency Pattern, one type of CM DPs, is lower than that of normal people, reflecting that melatonin has a certain correlation with DP [37]. In our study, the level of 5-Methoxytryptamine is lower than normal in RA DP. Simultaneously, Melatonin Degradation II and the Superpathway of Melatonin Degradation have been confirmed to be closely related to RA DP by bioinformatics analysis.

GABA, one of the specific metabolites of RA EP, showed a decreasing trend. GABA is the main inhibitory neurotransmitter in the brain, mainly involved in cell proliferation, differentiation and migration [38]. GABA is mainly synthesized by glutamic acid via glutamic acid decarboxylase and acts through electrophilic GABA (A) receptors and/or hyper electrophilic GABA (B) receptors [39]. In neurological diseases, GABA has a good anticonvulsant effect and can be used to fight the occurrence of epilepsy, by inhibiting the sudden abnormal firing of neurons in the brain, thereby normalizing brain dysfunction [40]. The increase of GABA can alleviate the empirical pathological state, in other words, the decrease of GABA will aggravate the empirical pathological state in the body. The level of GABA decreased in RA EP, which reflected that the inhibition of neurotransmitters was reduced and bodily functions are more active than normal state in patients with RA EP.

The results of this study indicate that metabolomics can provide a basis for the classification of RA DP and EP. In other words, the classification of CM patterns can be distinguished by the difference of metabolites. The biological basis of the classification of RA DP and EP provides a new insight into the modern scientific connotation of RA, which will be helpful for the personalized treatment of RA patients. However, there are still some limitations in this study: (1) The sample size of this study was small, but representative RA DP and EP samples were still collected based on the rigorous diagnostic criteria, inclusion criteria and exclusion criteria, and meaningful results had been obtained; (2) The participants included in the study were all from Henan Province, which might reduce the influence of environmental factors on traditional medicine patterns of RA to some extent. However, the results could also reflect the traditional medicine patterns of RA in central China. Future studies under different regions and environments are still needed; (3) Since there is no animal and cell models of DP and EP, the study did not verify the findings based on cell biology experiments. However, metabolomics and bioinformatics analysis found some interesting results, which can provide a scientific basis for the biological basis of RA DP and EP and thoughts for further study of traditional medicine patterns of ICD-11; (4) DP and EP are the basic steps of the principle-based patterns for the diagnosis of RA. Subgroups can still be classified under each CM pattern diagnosis. For example, liver and kidney deficiency syndrome is one of the important subgroups of DP, while cold and dampness syndrome is one of the important subgroups of EP, which needs to be further studied.

## Conclusions

In conclusion, serum metabolomics preliminarily revealed the biological basis of RA DP and EP. 5-methoxytryptamine, LysoPC(18:1(9Z)/0:0) and GABA, LysoPA(18:1(9Z)/0:0), L-Targinine might be the predictors to distinguish the DP and EP of RA respectively, which provides a potential basis for the understanding of CM pattern diagnoses. These interesting results provide thoughts for further study of traditional medicine patterns of ICD-11. It also contributes to provide strategy for personalized precision treatment of RA and further validation is needed.

## Data Availability

The datasets generated and/or analyzed during the current study are available from the corresponding author upon reasonable request.

## Abbreviations

RA: Rheumatoid arthritis
CM: Chinese medicine
DP: Deficiency pattern
EP: Excess pattern
ICD-11: International Classification of Diseases 11th Revision
DM: Diabetes mellitus
MVA: Multivariable statistical analysis
PCA: Principal component
PLS-DA: Partial least squares discriminant
OPLS-DA: Orthogonal partial least square-discriminan.
VIP: Variable importance in the projection
RSD: Relative standard deviation
IPA: Ingenuity Pathway Analysis system
BMI: Body mass index
WBC: White blood cell
RBC: Red blood cell
HGB: Hemoglobin
PLT: Platelet
ALB: Albumin
ESR: Erythrocyte sedimentation rate
CRP: C-reactive protein
RF: Rheumatoid factor
LysoPA: Lysophosphatidic acid
LysoPE: Lysophosphatidylethanolamine
LysoPC: Lysophosphatidylcholine
DG: Diacyl glycerol
PE: Phosphatidylethanolamine
PC: Phosphatidylcholine
GABA: Gamma-Aminobutyric acid
